# ﻿New records and revised distribution of tiger beetles in China (Coleoptera, Cicindelidae)

**DOI:** 10.3897/zookeys.1210.127753

**Published:** 2024-08-15

**Authors:** Ke-Yi Wang, Fabian A. Boetzl, Jürgen Wiesner, Cheng-De Li

**Affiliations:** 1 School of Forestry, Northeast Forestry University, Harbin, 150040, China Northeast Forestry University Harbin China; 2 Department of Ecology, Swedish University of Agricultural Sciences, Uppsala SE-750 07, Sweden Swedish University of Agricultural Sciences Uppsala Sweden; 3 Am Zellberg 6, D-38527 Meine, Germany Unaffiliated Meine Germany

**Keywords:** China, distribution, new records, taxonomy, tiger beetles

## Abstract

Based on the examination of specimens housed in several scientific collections, we expand the known tiger beetle fauna of China, and eight species are recorded from China for the first time. The occurrence of Cicindela (Cicindela) sachalinensis raddei Morawitz, 1863 in Shanxi Province, and Neocollyris (Neocollyris) saphyrina (Chaudoir, 1850) in China are re-established. We provide distribution maps and habitus photographs of examined specimens for the newly recorded and revised species. We also discuss potential research hotspots for future taxonomic studies of tiger beetles in China.

## ﻿Introduction

Tiger beetles (Coleoptera, Cicindelidae) are a globally distributed family that occupies almost all terrestrial ecosystems (Pearson and Vogler 2001). More than 2,850 species are currently known to science (Wiesner 2020), of which the richest diversity is found in the Oriental realm (Cassola and Pearson 2000; Pearson and Wiesner 2023). China spans both the Palearctic and Oriental realms, with significant climatic differences both between north and south as well as between west and east. In addition, altitudinal zones should be also considered. It features numerous examples of biogeographic isolation that can facilitate speciation. These biogeographic barriers include the Qinling Mountains–Huaihe River, Hengduan Mountains, Tianshan Mountains, Greater Khingan Mountains, Nanling Mountains, and so on. As a result, China has a unique and rich tiger beetle fauna, representing a mixture of Palearctic and Oriental species together with a high degree of endemism (Li et al. 2012).

There has been limited research on the overall distribution of tiger beetles in China (Shook and Wiesner 2006; Wu and Shook 2007; Wu 2011; Aston 2016, 2018). The distribution data that have been available was primarily sourced from publications, private specimen collections, and local museums. Consequently, the known distributional ranges of some species in China are patchy and the actual ranges of these species may extend far beyond the existing records.

This article summarizes data from tiger beetle specimens in several universities and research institutes across China, along with those collected by the corresponding author’s research team. Through these specimens, we update and expand the known distribution of some species of tiger beetle throughout China. Some provinces included here have lacked new records of tiger beetles for many years. The results expand our understanding of the tiger beetle fauna of China.

## ﻿Materials and methods

In addition to these specimen data, other records were extracted from Wiesner (2020) and Lorenz (2021).

Photographs of the habitus of specimens were taken with a Canon EOS M6 Mark II. A Godox TT350c Flash was used as a light source. Helicon Focus v. 7 was used for image stacking and all images were further processed in Adobe Photoshop CS6.

The specimens reported below are deposited in the following collections:

**EMCAU**Entomological Museum of China Agricultural University, Beijing, China

**IZCAS**Institute of Zoology, Chinese Academy of Sciences, Beijing, China

**KIZCAS**Kunming Institute of Zoology, Chinese Academy of Sciences, Kunming, China

**NEFU** Northeast Forestry University, Harbin, China

**Figure 1.**
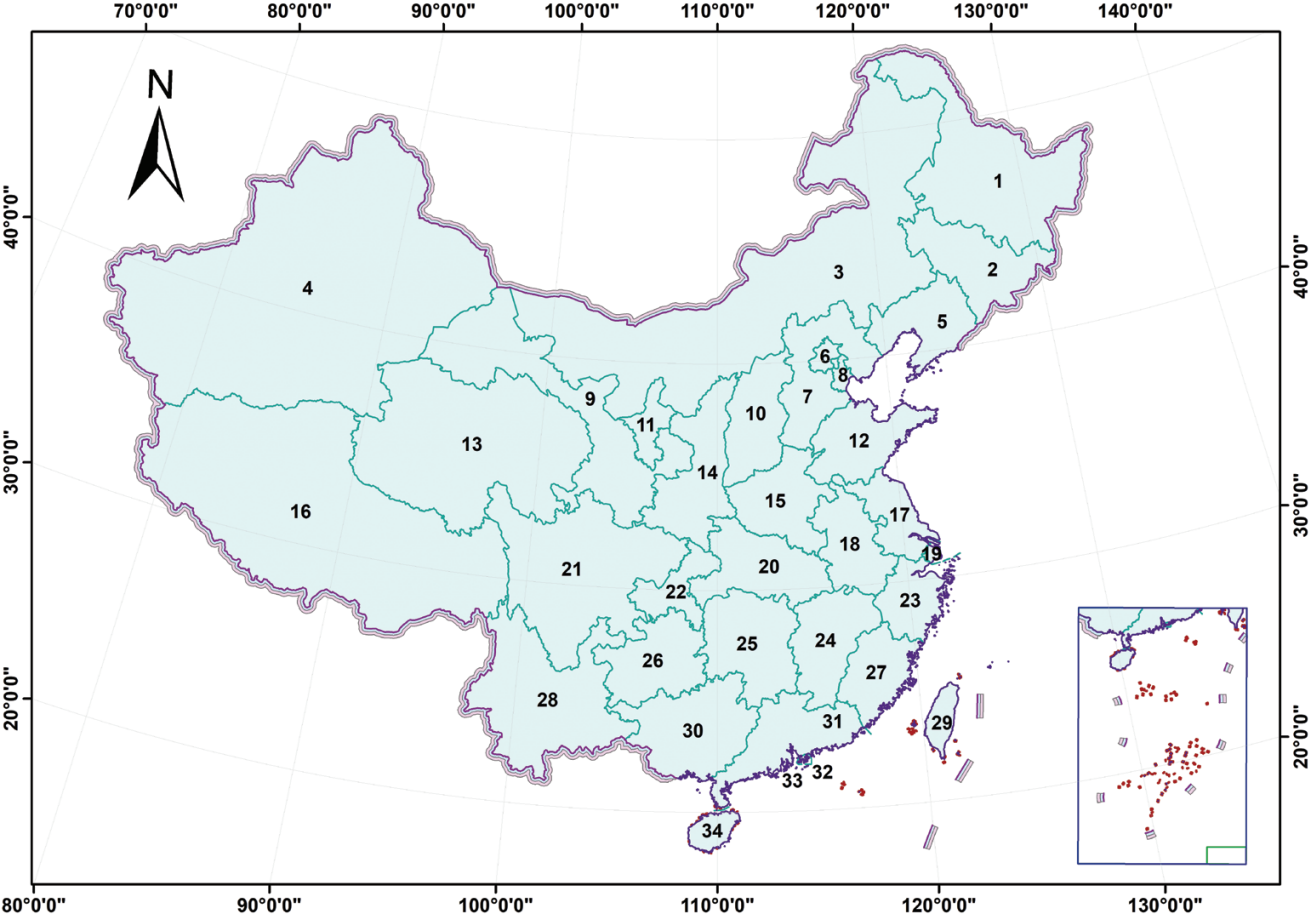
Map of the 34 provinces, autonomous regions, and municipalities of China (see Table 1).

Distribution maps were generated using ArcGIS v. 10.2. Map data were sourced online from http://xzqh.mca.gov.cn/map (Fig. 1). The English translations of Chinese provinces and regions were partially extracted from https://www.stats.gov.cn/sj/ndsj/2023/indexeh.htm (Table 1). Distribution maps of species were processed using Adobe Photoshop CS6.

**Table 1.** Provinces, autonomous regions, and municipalities of China.

1.	Heilongjiang	2.	Jilin
3.	Inner Mongolia	4.	Xinjiang
5.	Liaoning	6.	Beijing
7.	Hebei	8.	Tianjin
9.	Gansu	10.	Shanxi
11.	Ningxia	12.	Shandong
13.	Qinghai	14.	Shaanxi
15.	Henan	16.	Xizang
17.	Jiangsu	18.	Anhui
19.	Shanghai	20.	Hubei
21.	Sichuan	22.	Chongqing
23.	Zhejiang	24.	Jiangxi
25.	Hunan	26.	Guizhou
27.	Fujian	28.	Yunnan
29.	Taiwan	30.	Guangxi
31.	Guangdong	32.	Hong Kong
33.	Macao	34.	Hainan

## ﻿Taxonomy

**Family Cicindelidae Latreille, 1802**

**Tribe Cicindelini Latreille, 1802**

**Subtribe Cicindelina Latreille, 1802**

### Genus *Abroscelis* Hope, 1838

#### ﻿ Abroscelis anchoralis anchoralis

Taxon classificationAnimaliaColeopteraCicindelidae

(Chevrolat, 1845)

69FBE1B2-970E-5779-9F0E-26AF250BA491

Figs 2A , 3A

Cicindela anchoralis : Chevrolat 1845: 7.
Abroscelis anchoralis anchoralis : Lin and Ho 2007: 180; Shook and Wiesner 2006: 7; Wu 2011: 23.

##### Published data.

Liaoning (Shook and Wiesner 2006: 7; Wu 2011: 23), Beijing (Shook and Wiesner 2006: 7; Wu 2011: 23), Hebei (Shook and Wiesner 2006: 7), Shandong (Shook and Wiesner 2006: 7; Wu 2011: 23), Zhejiang (Shook and Wiesner 2006: 7; Wu 2011: 23), Hainan (Lin and Ho 2007: 180), Hong Kong (Shook and Wiesner 2006: 7; Wu 2011: 23), Macao (Chevrolat 1845: 7; Shook and Wiesner 2006: 7; Wu 2011: 23), Taiwan (Lin and Ho 2007: 180).

**Figure 2.**
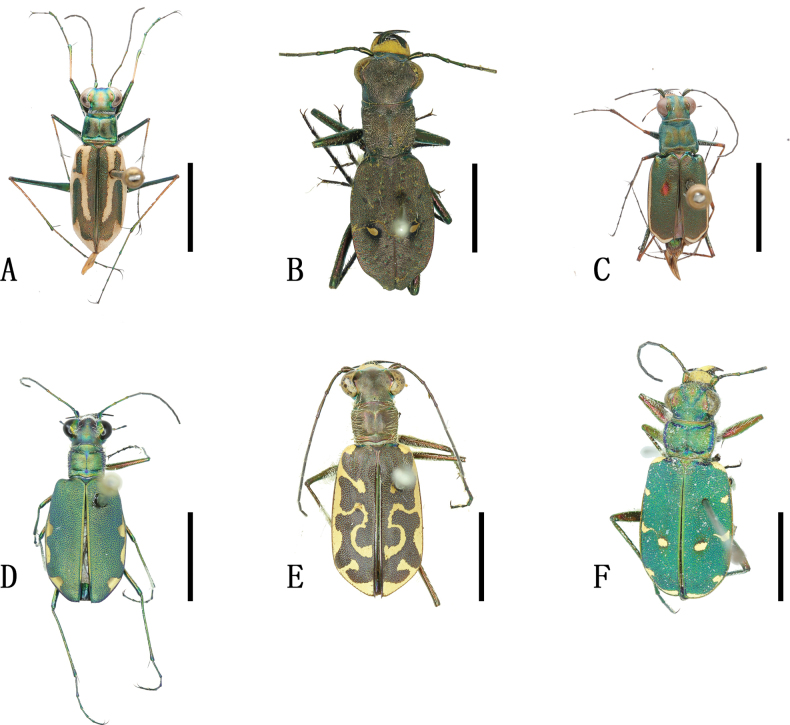
Habitus photographs **A***Abroscelisanchoralisanchoralis* (Chevrolat, 1845) **B**Apterodela (Apterodela) bivirgulata bivirgulata (Fairmaire, 1889) **C***Callytronnivicinctum* (Chevrolat, 1845) **D***Calomerachloris* (Hope, 1831) **E***Calomeraplumigerascoliographa* (Rivalier, 1953) **F**Cicindela (Cicindela) campestris pontica Fischer, 1828. Scale bars: 5 mm.

##### New records.

Guangdong, Zhanjiang, Min’an Town, 20°57'28"N, 110°15'6"E, 1 m, leg. H.B. Liang and X.L. Huang, 28.vi.2014, 1 male (IZCAS).

**Figure 3.**
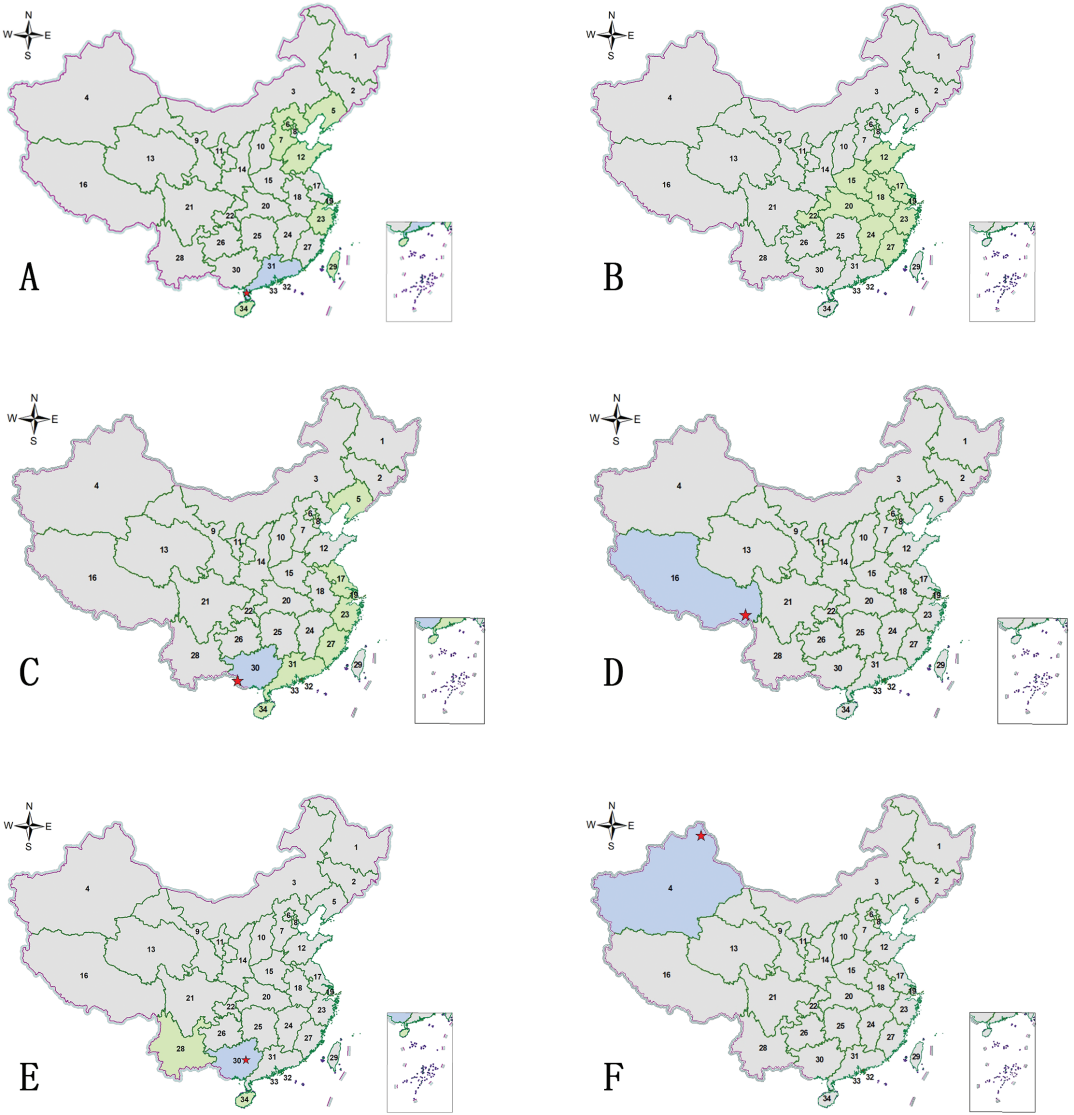
Distribution maps. Green indicates records with previously known distribution based on published data, blue indicates new records, red stars indicate the collection sites of the examined specimens **A***Abroscelisanchoralisanchoralis* (Chevrolat, 1845) **B**Apterodela (Apterodela) bivirgulata bivirgulata (Fairmaire, 1889) **C***Callytronnivicinctum* (Chevrolat, 1845) **D***Calomerachloris* (Hope, 1831) **E***Calomeraplumigerascoliographa* (Rivalier, 1953) **F**Cicindela (Cicindela) campestris pontica Fischer, 1828.

##### Distribution.

China (Guangdong, Liaoning, Beijing, Hebei, Shandong, ?Jiangsu, Zhejiang, ?Fujian, Hainan, Hong Kong, Macao, Taiwan).

##### Remarks.

New provincial record for Guangdong.

### ﻿Genus *Apterodela* Rivalier, 1950

**Subgenus Apterodela Rivalier, 1950**

#### ﻿ Apterodela (Apterodela) bivirgulatabivirgulata

Taxon classificationAnimaliaColeopteraCicindelidae

(Fairmaire, 1889)

6C9D0FCA-9EC4-5EC7-ACF8-E74B06BC577B

Figs 2B , 3B

Cicindela bivirgulata : Fairmaire 1889: 5.
Cylindera (Apterodela) lobipennis : Shook and Wiesner 2006: 13; Wu 2011: 26.
Apterodela (Apterodela) bivirgulata : Matalin et al. 2024: 311.

##### Published data.

Shandong (Matalin et al. 2024: 311), Henan (Matalin et al. 2024: 311), Hubei (Matalin et al. 2024: 311), Anhui (Matalin et al. 2024: 311), Jiangxi (Matalin et al. 2024: 311), Jiangsu (Matalin et al. 2024: 311), Shanghai (Matalin et al. 2024: 311), Chongqing (Matalin et al. 2024: 311), Zhejiang (Matalin et al. 2024: 311), Fujian (Matalin et al. 2024: 311).

##### Records.

Jiangsu, leg. unknown, 18.iv.1923, 1 male (IZCAS).

##### Distribution.

China (Shandong, Henan, Hubei, Anhui, Jiangxi, Jiangsu, Shanghai, Chongqing, Zhejiang, Fujian).

##### Remarks.

Additional specimens contribute to a better understanding of the distribution and a more robust identification of A. (A.) b. bivirgulata.

### ﻿Genus *Callytron* Gistl, 1848

#### ﻿ Callytron nivicinctum

Taxon classificationAnimaliaColeopteraCicindelidae

(Chevrolat, 1845)

F2BB331D-1CAB-5C72-A248-DD50B10B8983

Figs 2C , 3C

Cicindela nivicincta : Chevrolat 1845: 98.
Callytron nivicinctum : Shook and Wiesner 2006: 8; Wu 2011: 23; Wiesner et al. 2017: 68.

##### Published data.

Liaoning (Wu 2011: 23; Wiesner et al. 2017: 68), Jiangsu (Shook and Wiesner 2006: 8; Wu 2011: 23; Wiesner et al. 2017: 68), Zhejiang (Shook and Wiesner 2006: 8; Wu 2011: 23; Wiesner et al. 2017: 68), Shanghai (Shook and Wiesner 2006: 8; Wu 2011: 23; Wiesner et al. 2017: 68), Fujian (Shook and Wiesner 2006: 8; Wu 2011: 23; Wiesner et al. 2017: 68), Guangdong (Shook and Wiesner 2006: 8; Wu 2011: 23; Wiesner et al. 2017: 68), Hainan (Shook and Wiesner 2006: 8; Wu 2011: 23; Wiesner et al. 2017: 68), Hong Kong (Shook and Wiesner 2006: 8; Wu 2011: 23; Wiesner et al. 2017: 68), Macao (Shook and Wiesner 2006: 8; Wu 2011: 23; Wiesner et al. 2017: 68).

##### New records.

Guangxi, Longzhou, Mount Daqing, leg. J.K. Yang, 15.v.1963, 1 female (EMCAU).

##### Distribution.

China (Guangxi, Liaoning, Jiangsu, Zhejiang, Shanghai, Fujian, Guangdong, Hainan, Hong Kong, Macao), South Korea, Japan, Cambodia, Vietnam.

##### Remarks.

New provincial record for Guangxi.

### ﻿Genus *Calomera* Motschulsky, 1862

#### ﻿ Calomera chloris

Taxon classificationAnimaliaColeopteraCicindelidae

(Hope, 1831)

6D9E9849-A274-5F62-8623-506D621B6C3A

Figs 2D , 3D

Cicindela chloris : Hope 1831: 21.

##### New records.

Xizang, Linzhi, Xiachayu, 28°29'59"N, 97°1'3"E, leg. J. Wu, 28.vii.2014, 1 female (IZCAS); Xizang, Bomi, Tongmai, 2250 m, leg. unknown, 31.viii.2005, 1 female (IZCAS).

##### Distribution.

China (Xizang), Afghanistan, Pakistan, Nepal, Bhutan, India, Laos.

##### Remarks.

New state record for China and new provincial record for Xizang.

#### ﻿ Calomera plumigera scoliographa

Taxon classificationAnimaliaColeopteraCicindelidae

(Rivalier, 1953)

96E83B43-DBC6-501A-AABC-A7B69D9CF43C

Figs 2E , 3E

Calomera plumigera scoliographa : Shook and Wiesner 2006: 9; Shook and Wu 2006: 39; Wu 2011: 24.

##### Published data.

Yunnan (Shook and Wiesner 2006: 9; Shook and Wu 2006: 39; Wu 2011: 24), Hainan (Shook and Wiesner 2006: 9; Wu 2011: 24).

##### New records.

Guangxi, Jinxiu, Dazhang, leg. unknown, ?.v.1976, 1 male (IZCAS).

##### Distribution.

China (Guangxi, Yunnan, Hainan), Vietnam, Laos, Cambodia, Malaysia, Thailand.

##### Remarks.

New provincial record for Guangxi.

### ﻿Genus *Cicindela* Linnaeus, 1758

**Subgenus Cicindela Linnaeus, 1758**

#### ﻿ Cicindela (Cicindela) campestrispontica

Taxon classificationAnimaliaColeopteraCicindelidae

Fischer, 1828

588526A5-3001-5B11-AEA0-5EDBBCA53DD1

Figs 2F , 3F

Cicindela campestris pontica : Gebert et al. 2021: 473.

##### New records.

Xinjiang, Altai, Forest Region of Ashan Forest Management Bureau, leg. unknown, v.1981, 1 female (IZCAS).

##### Distribution.

China (Xinjiang), Ukraine, Bulgaria, Turkey, Georgia, Azerbaijan, Kazakhstan, Russia.

##### Remarks.

New state record for China and new provincial record for Xinjiang.

#### ﻿ Cicindela (Cicindela) sachalinensisraddei

Taxon classificationAnimaliaColeopteraCicindelidae

Morawitz, 1863

FAE76E01-7279-5DF2-85E1-FECD44844274

Figs 4A , 5A

Cicindela raddei : Morawitz 1863: 237, 238.
Cicindela sachalinensis : Yu and Wang 2017:145; Di and Ren 2021: 146.
Cicindela sachalinensis raddei : Mandl 1981: 27.
Cicindela (Cicindela) sachalinensis raddei : Shook and Wiesner 2006: 11; Wu 2011: 25.

##### Published data.

Heilongjiang (Shook and Wiesner 2006: 11; Wu 2011: 25), Gansu (Shook and Wiesner 2006: 11; Wu 2011: 25), Beijing (Huairou) (Yu and Wang 2017: 145), Hebei (Mount Xiaowutai) (Di and Ren 2021: 146), Qinghai (Shook and Wiesner 2006: 11; Wu 2011: 25), Shanxi (Wutaishan) (Mandl 1981: 27), Hubei (Shook and Wiesner 2006: 11; Wu 2011: 25), Sichuan (Shook and Wiesner 2006: 11; Wu 2011: 25).

**Figure 4.**
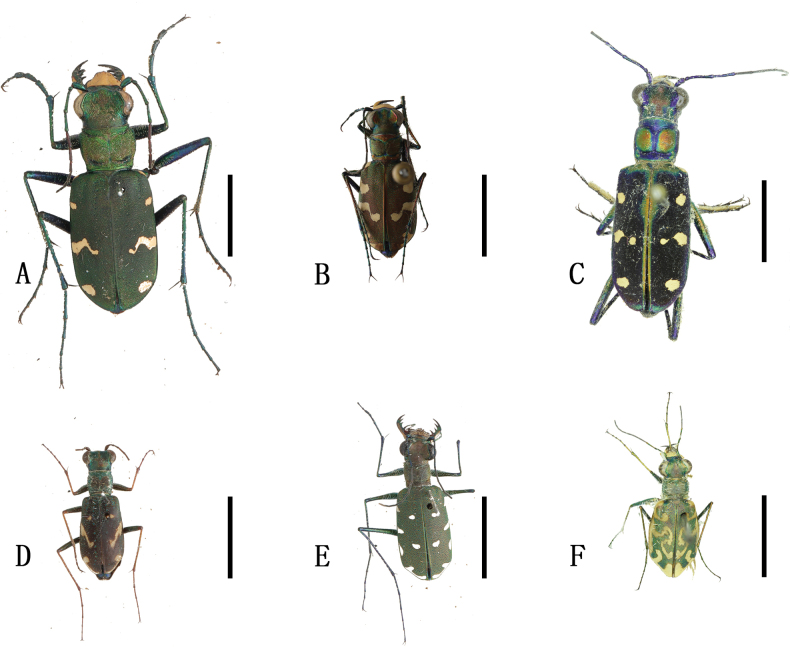
Habitus photographs **A**Cicindela (Cicindela) sachalinensis raddei Morawitz, 1863 **B**Cicindela (Cicindela) transbaicalica hamifasciata Kolbe, 1886 **C***Cosmodelaseparata* (Fleutiaux, 1893) **D**Cylindera (Cylindera) obliquefasciata (Adams, 1817) **E**Cylindera (Eriodera) albopunctata (Chaudoir, 1852) **F**Cylindera (Eugrapha) contorta contorta (Fischer, 1828). Scale bars: 5 mm.

##### Records.

Shanxi, Mount Wutai, Dongtai, 2400 m, leg. T.S. Li, 13.vi.1964, 1 female (IZCAS).

**Figure 5.**
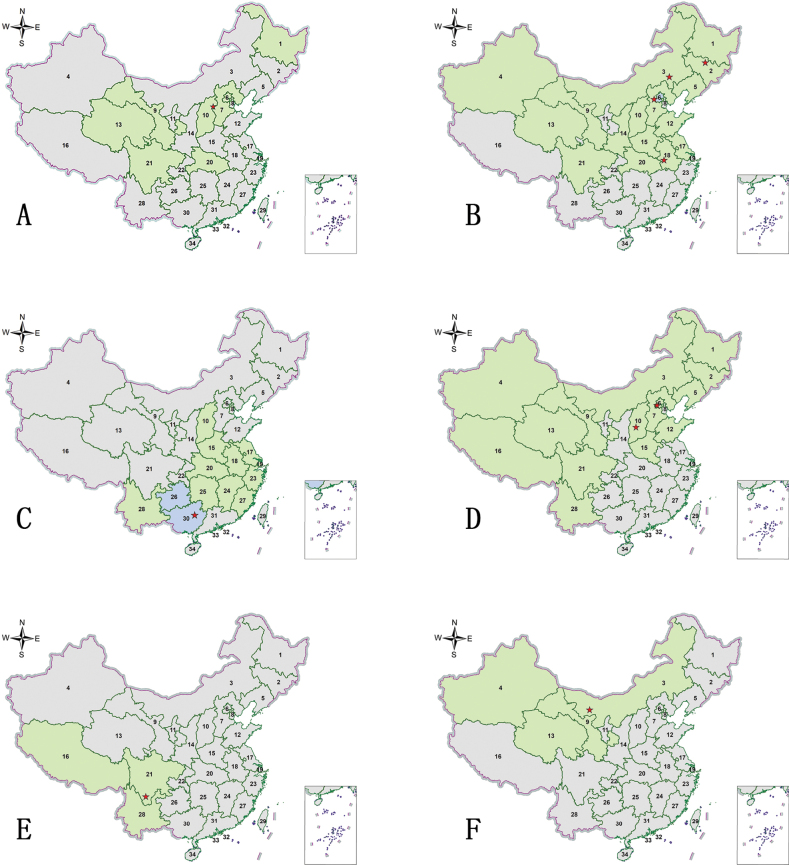
Distribution maps. Green indicates records with previously known distribution based on published data, blue indicates new records, red stars indicate the collection sites of the examined specimens **A**Cicindela (Cicindela) sachalinensis raddei Morawitz, 1863 **B**Cicindela (Cicindela) transbaicalica hamifasciata Kolbe, 1886 **C***Cosmodelaseparata* (Fleutiaux, 1893) **D**Cylindera (Cylindera) obliquefasciata (Adams, 1817) **E**Cylindera (Eriodera) albopunctata (Chaudoir, 1852) **F**Cylindera (Eugrapha) contorta contorta (Fischer, 1828).

##### Distribution.

China (Shanxi, Heilongjiang, Gansu, Qinghai, Beijing, Hebei, Hubei, Sichuan), Russia, Mongolia.

##### Remarks.

Mandl (1981) reported C. (C.) s. raddei from Chunshantu, Mount Wutai, Shanxi. This record was not accepted in previous checklists. We restore the provincial record of C. (C.) s. raddei for Shanxi.

#### ﻿ Cicindela (Cicindela) transbaicalicahamifasciata

Taxon classificationAnimaliaColeopteraCicindelidae

Kolbe, 1886

5A88E824-7392-5BFD-B022-4CF943C8190B

Figs 4B , 5B

Cicindela japanensis hamifasciata : Kolbe 1886: 170.
Cicindela hybrida transbaicalica : Wang et al. 2012: 15.
Cicindela (Cicindela) transbaicalica hamifasciata : Shook and Wiesner 2006: 11; Wu 2011: 25.

##### Published data.

Heilongjiang (Wu 2011: 25), Jilin (Wu 2011: 25), Inner Mongolia (Wu 2011: 25), Liaoning (Wu 2011: 25; Wang et al. 2012: 15), Gansu (Shook and Wiesner 2006: 11; Wu 2011: 25), Xinjiang (Wu 2011: 25), Shaanxi (Shook and Wiesner 2006: 11; Wu 2011: 25), Shanxi (Wu 2011: 25), Anhui (Wu 2011: 25), Henan (Shook and Wiesner 2006: 11; Wu 2011: 25), Hebei (Shook and Wiesner 2006: 11; Wu 2011: 25), Hubei (Wu 2011: 25), Jiangsu (Wu 2011: 25), Shandong (Shook and Wiesner 2006: 11; Wu 2011: 25), Sichuan (Shook and Wiesner 2006: 11; Wu 2011: 25), Qinghai (Shook and Wiesner 2006: 11; Wu 2011: 25), Fujian (Shook and Wiesner 2006: 11; Wu 2011: 25), Guangdong (Wu 2011: 25), Zhejiang (Wu 2011: 25), ? Yunnan (Wu 2011: 25).

##### New records.

Beijing, Shunyi, leg. unknown, 13.v.1964, 2 males, 3 females (EMCAU); Jilin, Songhua River, Wukeshu, 44°27'43"N, 126°49'33"E, 200 m, leg. Y. Liu, 1.vi.2009, 1 male (IZCAS); Inner Mongolia, Chifeng, day collecting, leg. Y. Wang and S.Y. Geng, 26–30.vii. 2009, 1 female (NEFU); Anhui, Yuexi, Yaoluoping, 30°59'01"N, 116°05'10"E, 1178 m, leg. unknown, 17.v.2021, 1 female (IZCAS).

##### Distribution.

China (Beijing, Heilongjiang, Jilin, Inner Mongolia, Liaoning, Gansu, Xinjiang, Shaanxi, Shanxi, Anhui, Henan, Hebei, Hubei, Jiangsu, Shandong, Sichuan, Qinghai, ?Fujian, ?Guangdong, ?Zhejiang, ?Yunnan), North Korea, South Korea, Russia.

##### Remarks.

New provincial record for Beijing. Wu (2011) expanded the known distribution of C. (C.) t. hamifasciata Kolbe, 1886 in China by reviewing the Chinese literature, but Wu did not provide specimen data. We provide specimen data for this species from Jilin, Inner Mongolia and Anhui for the first time. Since C. (C.) t. hamifasciata Kolbe, 1886 is a typical Eastern Palearctic species, we believe previous records from Fujian, Zhejiang, Guangdong, and Yunnan require further confirmation.

### ﻿Genus *Cosmodela* Rivalier, 1961

#### ﻿ Cosmodela separata

Taxon classificationAnimaliaColeopteraCicindelidae

(Fleutiaux, 1893)

4B69810B-ABE4-57FF-993B-592B5B991920

Figs 4C , 5C

Cicindela separata : Fleutiaux 1893: 491.
Cosmodela separata : Shook and Wiesner 2006: 13; Wu 2011: 26; Wiesner et al. 2017: 53; Tu et al. 2020: 68.

##### Published data.

Shanxi (Shook and Wiesner 2006: 13; Wu 2011: 26; Wiesner et al. 2017: 53), Jiangsu (Shook and Wiesner 2006: 13; Wu 2011: 26; Wiesner et al. 2017: 53), Shanghai (Fleutiaux 1893: 491; Shook and Wiesner 2006: 13; Wu 2011: 26; Wiesner et al. 2017: 53), Zhejiang (Fleutiaux 1893: 491; Shook and Wiesner 2006: 13; Wu 2011: 26; Wiesner et al. 2017: 53), Henan (Shook and Wiesner 2006: 13; Wu 2011: 26; Wiesner et al. 2017: 53), Anhui (Shook and Wiesner 2006: 13; Wu 2011: 26; Wiesner et al. 2017: 53), Hubei (Wu 2011: 26; Wiesner et al. 2017: 53), Hunan (Wu 2011: 26; Wiesner et al. 2017: 53), Jiangxi (Tu et al. 2020: 68), Fujian (Shook and Wiesner 2006: 13; Wu 2011: 26; Wiesner et al. 2017: 53), Yunnan (Shook and Wiesner 2006: 13; Wu 2011: 26; Wiesner et al. 2017: 53).

##### New records.

Guangxi, Jinxiu, Tongmu Town, leg. J.C. Huang, 4.vii.1981, 2 females (IZCAS); Guizhou, Shiqian, Mount Foding, 750 m, leg. X.K. Yang, 24.XI.1988, 1 female (IZCAS).

##### Distribution.

China (Guangxi, Guizhou, Shanxi, Jiangsu, Shanghai, Zhejiang, Henan, Anhui, Hubei, Hunan, Jiangxi, Fujian, Yunnan), Vietnam.

##### Remarks.

New provincial records for Guangxi and Guizhou.

### ﻿Genus *Cylindera* Westwood, 1831

**Subgenus Cylindera Westwood, 1831**

#### ﻿ Cylindera (Cylindera) obliquefasciata

Taxon classificationAnimaliaColeopteraCicindelidae

(Adams, 1817)

C902239F-BC28-5849-B2F3-2CBE335FB0CD

Figs 4D , 5D

Cicindela obliquefasciata : Adams 1817: 280.
Cylindera (Cylindera) obliquefasciata obliquefasciata : Shook and Wiesner 2006: 14; Wu 2011: 27.

##### Published data.

Heilongjiang (Shook and Wiesner 2006: 14; Wu 2011: 27), Jilin (Wu 2011: 27), Inner Mongolia (Shook and Wiesner 2006: 14; Wu 2011: 27), Liaoning (Wu 2011: 27), Beijing (Wu 2011: 27), Hebei (Shook and Wiesner 2006: 14; Wu 2011: 27), Gansu (Shook and Wiesner 2006: 14; Wu 2011: 27), Qinghai (Shook and Wiesner 2006: 14; Wu 2011: 27), Xinjiang (Shook and Wiesner 2006: 14; Wu 2011: 27), Shanxi (Wu 2011: 27), Shandong (Shook and Wiesner 2006: 14; Wu 2011: 27), Henan (Shook and Wiesner 2006: 14; Wu 2011: 27).

##### Records.

Beijing, Haidian, Fragrant Hills Park, leg. unknown, 16.vii.1962, 2 males, 3 females (EMCAU); Shanxi, Taiyuan, Qingxu, leg. unknown, 20.vi.1960, 1 male (EMCAU).

##### Distribution.

China (Heilongjiang, Jilin, Inner Mongolia, Liaoning, Beijing, Hebei, Gansu, Qinghai, Xinjiang, Shanxi, Shandong, Henan), Russia.

##### Remarks.

Wu (2011) reported C. (C.) obliquefasciata (Adams, 1817) from Beijing and Shanxi but did not provide specific specimen data. We provide specimen data for this species from Beijing and Sichuan for the first time.

### Subgenus ﻿Eriodera Rivalier, 1961

#### ﻿ Cylindera (Eriodera) albopunctata

Taxon classificationAnimaliaColeopteraCicindelidae

(Chaudoir, 1852)

4E4F5E6F-FB7A-5816-829B-87E6D9FCACC6

Figs 4E , 5E

Cicindela albopunctata : Chaudoir 1852: 10.
Cylindera (Eriodera) albopunctata : Shook and Wiesner 2006: 14.

##### Published data.

Sichuan (Wu 2011: 27), Yunnan (Shook and Wiesner 2006: 14; Wu 2011: 27), Xizang (Shook and Wiesner 2006: 14; Wu 2011: 27).

##### Records.

Sichuan, Yanyuan, Jinhe, 1200 m, leg. D.J. Liu, 29.vi.1984, 1 female (IZCAS).

##### Distribution.

China (Sichuan, Yunnan, Xizang), Pakistan, Nepal, Bhutan, India, Vietnam.

##### Remarks.

Wu (2011) reported C. (E.) albopunctata (Chaudoir, 1852) from Sichuan but did not provide specific specimen data. We provide specimen data for this species from Sichuan for the first time.

### ﻿Subgenus Eugrapha Rivalier, 1950

#### ﻿ Cylindera (Eugrapha) contortacontorta

Taxon classificationAnimaliaColeopteraCicindelidae

(Fischer, 1828)

7B6973BA-EAE0-5D3E-8258-E103A89693CE

Figs 4F , 5F

Cylindera (Eugrapha) contorta contorta : Shook and Wiesner 2006: 14.

##### Published data.

Gansu (Li and Chen 1993; Shook and Wiesner 2006: 14; Wu 2011: 27), Qinghai (Li and Chen 1993; Shook and Wiesner 2006: 14; Wu 2011: 27), Xinjiang (Li and Chen 1993; Shook and Wiesner 2006: 14; Wu 2011: 27), Inner Mongolia (Wu 2011: 27).

##### Records.

Inner Mongolia, Alxa League, Alxa Right Banner, leg. Y.C. Lv, 12.vii.1986, 1 male, 1 female (IZCAS).

##### Distribution.

China (Inner Mongolia, Gansu, Qinghai, Xinjiang), Ukraine, Romania, Moldova, Iran, Georgia, Azerbaijan, Kazakhstan, Uzbekistan, Turkmenistan, Tadzhikistan, Afghanistan, Russia, Mongolia.

##### Remarks.

Wu (2011) reported C. (E.) c. contorta (Fischer, 1828) from Inner Mongolia but did not provide specific specimen data. We provide specimen data for this species from Inner Mongolia for the first time.

#### ﻿ Cylindera (Eugrapha) elisaeelisae

Taxon classificationAnimaliaColeopteraCicindelidae

(Motschulsky, 1859)

17A4AB43-C514-5762-8267-F14977568BDF

Figs 6A , 7A

Cicindela elisae : Motschulsky 1859: 487; Wang et al. 2012: 16.
Cylindera (Eugrapha) elisae elisae : Shook and Wiesner 2006: 15; Wu 2011: 27; Bai 2016: 29.

##### Published data.

Heilongjiang (Wu 2011: 27), Jilin (Wu 2011: 27), Inner Mongolia (Wu 2011: 27), Liaoning (Wang et al. 2012:16), Hebei (Shook and Wiesner 2006: 15, Wu 2011: 27), Shandong (Shook and Wiesner 2006: 15, Wu 2011: 27), Beijing (Shook and Wiesner 2006: 15, Wu 2011: 27), Shanxi (Shook and Wiesner 2006: 15, Wu 2011: 27), Gansu (Shook and Wiesner 2006: 15, Wu 2011: 27), Xinjiang (Wu 2011: 27), Anhui (Wu 2011: 27), Henan (Shook and Wiesner 2006: 15, Wu 2011: 27), Hubei (Shook and Wiesner 2006: 15, Wu 2011: 27), Hunan (Shook and Wiesner 2006: 15, Wu 2011: 27), Zhejiang (Shook and Wiesner 2006: 15, Wu 2011: 27), Guangdong (Shook and Wiesner 2006: 15, Wu 2011: 27), Hainan (Wu 2011: 27), Guangxi (Shook and Wiesner 2006: 15, Wu 2011: 27), Fujian (Shook and Wiesner 2006: 15), Shanghai (Shook and Wiesner 2006: 15, Wu 2011: 27), Jiangsu (Shook and Wiesner 2006: 15, Wu 2011: 27), Jiangxi (Shook and Wiesner 2006: 15, Wu 2011: 27), Qinghai (Wu 2011: 27, Bai 2016: 29), Sichuan (Shook and Wiesner 2006: 15, Wu 2011: 27), Yunnan (Shook and Wiesner 2006: 15, Shook and Wu 2006: 41, Wu 2011: 27), Xizang (Wu 2011: 27), Hong Kong (Shook and Wiesner 2006: 15), Taiwan (Shook and Wiesner 2006: 15, Wu 2011: 27).

**Figure 6.**
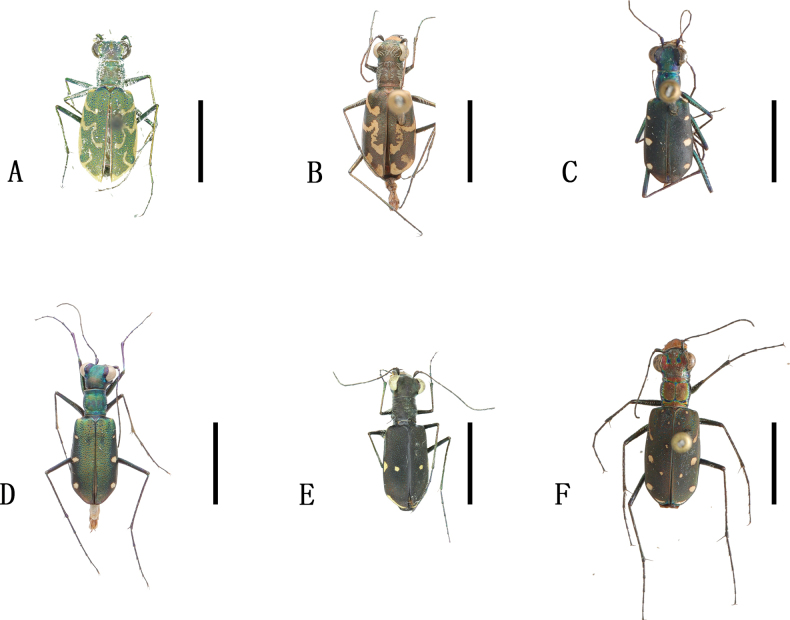
Habitus photographs **A**Cylindera (Eugrapha) elisae elisae (Motschulsky, 1859) **B**Cylindera (Eugrapha) sublacerata vicaria (Semenov, 1895) **C**Cylindera (Ifasina) decolorata (Horn, 1907) **D**Cylindera (Ifasina) lesnei (Babault, 1923) **E**Cylindera (Ifasina) sikhimensis (Mandl, 1982) **F**Lophyra (Spilodia) lineifrons (Chaudoir, 1865). Scale bars: 5 mm.

##### New records.

Heilongjiang, Harbin, Hadeng (哈灯, hand-written label), leg. unknown, 13.vii.1963, 2 males, 1 female (NEFU).

**Figure 7.**
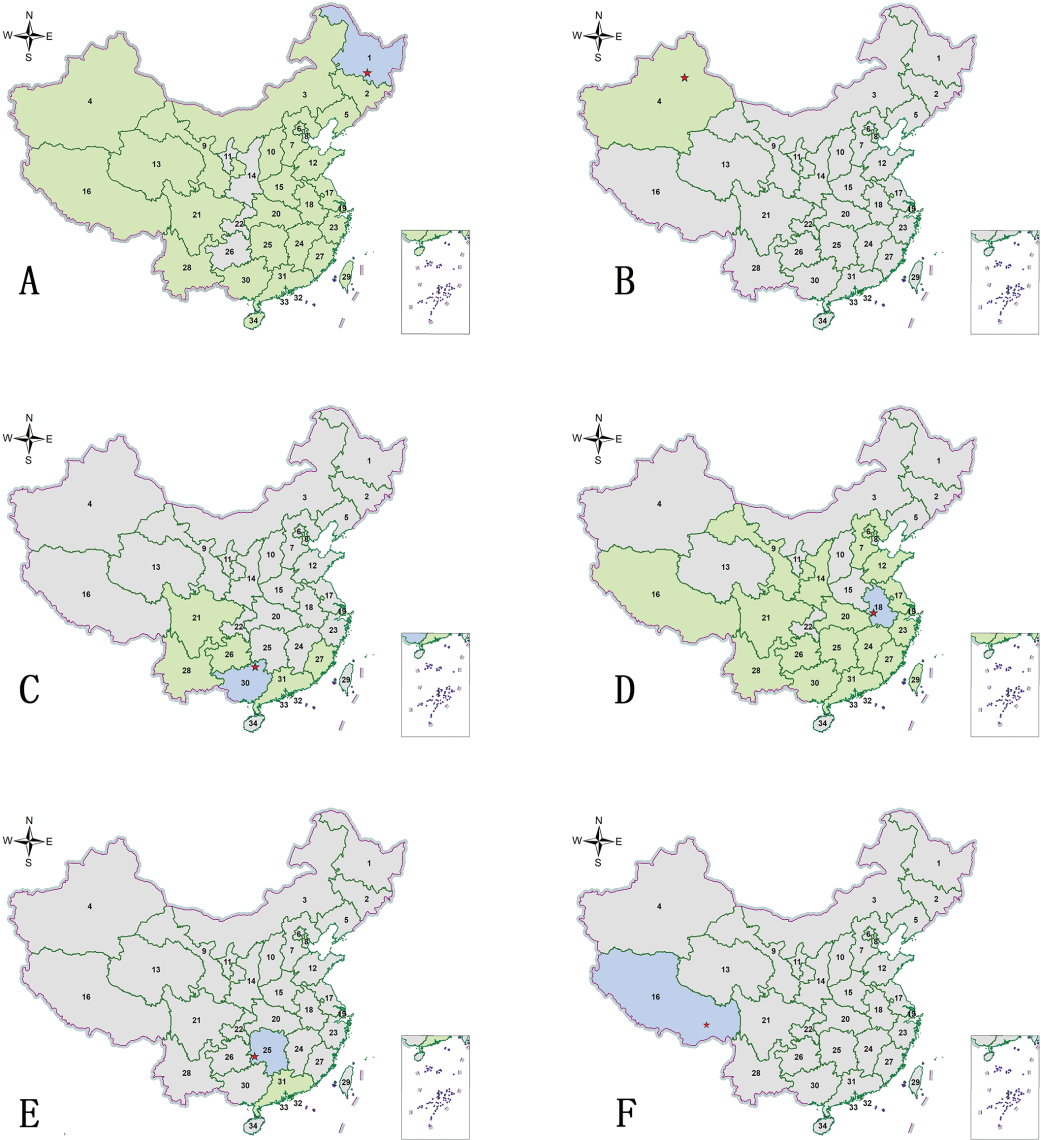
Distribution maps. Green indicates records with previously known distribution based on published data, blue indicates new records, red stars indicate the collection sites of the examined specimens **A**Cylindera (Eugrapha) elisae elisae (Motschulsky, 1859) **B**Cylindera (Eugrapha) sublacerata vicaria (Semenov, 1895) **C**Cylindera (Ifasina) decolorata (Horn, 1907) **D**Cylindera (Ifasina) kaleea kaleea (Bates, 1866) **E**Cylindera (Ifasina) lesnei (Babault, 1923) **F**Cylindera (Ifasina) sikhimensis (Mandl, 1982).

##### Distribution.

China (Heilongjiang, Jilin, Inner Mongolia, Liaoning, Hebei, Shandong, Beijing, Shanxi, Gansu, Xinjiang, Anhui, Henan, Hubei, Hunan, Zhejiang, Guangdong, Hainan, Guangxi, Fujian, Shanghai, Jiangsu, Jiangxi, Qinghai, Sichuan, Yunnan, Xizang, Hong Kong, Taiwan), North Korea, South Korea, Vietnam, Russia, Mongolia.

##### Remarks.

Wu (2011) and Wang et al. (2012) reported C. (E.) elisae from Heilongjiang without specimen record and detailed locality which makes it impossible to determine which subspecies was recorded. It is possible that C. (E.) e. hulunbeierensis is distributed in Heilongjiang as well. We hence provide the first record C. (E.) elisae for Heilongjiang.

#### ﻿ Cylindera (Eugrapha) sublaceratavicaria

Taxon classificationAnimaliaColeopteraCicindelidae

(Semenov, 1895)

7C254C97-1137-5FC5-923C-809F16558D09

Figs 6B , 7B

Cicindela sublacerata : Mandl 1955: 321.
Cicindela (Eugrapha) sublacerata : Acciavatti and Pearson 1989: 302.
Cylindera (Eugrapha) sublacerata vicaria : Wu 2011: 27.

##### Published data.

Xinjiang (Acciavatti and Pearson 1989: 303; Li and Chen 1993: 107; Wu 2011: 27).

##### Records.

Xinjiang, Changji, Fukang Desert Ecological System Observatary, 44°17'31"N, 87°56'3"E, 474 m, light trap, leg. Y. Liu, 11.vi.2007, 1 female (IZCAS).

##### Distribution.

China (Xinjiang), Mongolia.

##### Remarks.

We provide additional specimen data for this species from Xinjiang.

### Subgenus ﻿Ifasina Jeannel, 1946

#### ﻿ Cylindera (Ifasina) decolorata

Taxon classificationAnimaliaColeopteraCicindelidae

(Horn, 1907)

D6370D92-A002-5FBB-B63D-AB37BDFAF89C

Figs 6C , 7C

Cicindela psilica decolorata : Horn 1907: 24.
Cylindera (Ifasina) decolorate : Shook and Wiesner 2006: 15; Wu 2011: 27.

##### Published data.

Fujian (Shook and Wiesner 2006: 15; Wu 2011: 27), Guangdong (Shook and Wiesner 2006: 15; Wu 2011: 27), Sichuan (Shook and Wiesner 2006: 15; Wu 2011: 27), Guizhou (Shook and Wiesner 2006: 15; Wu 2011: 27), Yunnan (Horn 1907: 24; Shook and Wiesner 2006: 15; Wu 2011: 27).

##### New records.

Guangxi, Huaping Nature Reserve, Mount Tianping, leg. J.K. Yang, 5.vi.1963, 1 male, 1 female (EMCAU); Guangxi, Huaping Nature Reserve, Dayan Station, 25°36'52"N, 109°52'33"E, 1061 m, leg. M.Y. Lin and Y.Y. Qin, 12.vii.2022, 5 males, 18 females (IZCAS).

##### Distribution.

China (Guangxi, Fujian, Guangdong, Sichuan, Guizhou, Yunnan), Vietnam.

##### Remarks.

New provincial record for Guangxi.

#### ﻿ Cylindera (Ifasina) kaleeakaleea

Taxon classificationAnimaliaColeopteraCicindelidae

(Bates, 1866)

3884E831-2AAB-5B67-A4B7-4933C4ED9066

Fig. 7D

Cicindela kaleea : Bates 1866: 340, 341.
Cylindera (Ifasina) kaleea kaleea : Shook and Wiesner 2006: 15, 16; Wu 2011: 28.

##### Published data.

Beijing (Shook and Wiesner 2006: 15, 16; Wu 2011: 28), Hebei (Shook and Wiesner 2006: 15, 16; Wu 2011: 28), Shaanxi (Shook and Wiesner 2006: 15, 16; Wu 2011: 28), Gansu (Shook and Wiesner 2006: 15, 16; Wu 2011: 28), Shandong (Shook and Wiesner 2006: 15, 16; Wu 2011: 28), Jiangsu (Shook and Wiesner 2006: 15, 16; Wu 2011: 28), Shanghai (Shook and Wiesner 2006: 15, 16; Wu 2011: 28), Zhejiang (Shook and Wiesner 2006: 15, 16; Wu 2011: 28), Jiangxi (Shook and Wiesner 2006: 15, 16; Wu 2011: 28), Fujian (Shook and Wiesner 2006: 15, 16; Wu 2011: 28), Henan (Shook and Wiesner 2006: 15, 16; Wu 2011: 28), Hubei (Shook and Wiesner 2006: 15, 16; Wu 2011: 28), Hunan (Shook and Wiesner 2006: 15, 16; Wu 2011: 28), Guangdong (Shook and Wiesner 2006: 15, 16; Wu 2011: 28), Guangxi (Shook and Wiesner 2006: 15, 16; Wu 2011: 28), Sichuan (Shook and Wiesner 2006: 15, 16; Wu 2011: 28), Guizhou (Shook and Wiesner 2006: 15, 16; Wu 2011: 28), Yunnan (Shook and Wiesner 2006: 15, 16; Wu 2011: 28), Xizang (Shook and Wiesner 2006: 15, 16; Wu 2011: 28), Hong Kong (Shook and Wiesner 2006: 15, 16; Wu 2011: 28), Taiwan (Bates 1866: 340, 341; Shook and Wiesner 2006: 15, 16; Wu 2011: 28).

##### New records.

Anhui, Tiantangzhai, Tudiling Bridge, 31°30'23"N, 116°09'07"E, 635 m, leg. unknown, 24.ix.2021, 1 male (IZCAS).

##### Distribution.

China (Anhui, Beijing, Hebei, Shaanxi, Gansu, Shandong, Jiangsu, Shanghai, Zhejiang, Jiangxi, Fujian, Henan, Hubei, Hunan, Guangdong, Guangxi, Sichuan, Guizhou, Yunnan, Xizang, Hong Kong, Taiwan), India, Myanmar, Thailand, Laos, Vietnam.

##### Remarks.

New provincial record for Anhui.

#### ﻿ Cylindera (Ifasina) lesnei

Taxon classificationAnimaliaColeopteraCicindelidae

(Babault, 1923)

8837F080-1B57-51C6-8715-AF0DC9F8206B

Figs 6D , 7E

Cicindela lesnei : Babaule 1923: 7.
Cylindera (Ifasina) lesnei : Shook and Wiesner 2006: 16; Wu 2011: 28.

##### Published data.

Guangdong (Shook and Wiesner 2006: 16; Wu 2011: 28).

##### New records.

Hunan, Huitong, Raochong Village, 26°51'23"N, 109°50'43"E, 650 m, leg. H.B. Liang, 21.vi.2015, 1 female (IZCAS).

##### Distribution.

China (Hunan, Guangdong), Vietnam.

##### Remarks.

New provincial record for Hunan.

#### ﻿ Cylindera (Ifasina) sikhimensis

Taxon classificationAnimaliaColeopteraCicindelidae

(Mandl, 1982)

15C7AC5C-7815-5574-B867-D92C3B0138C6

Figs 6E , 7F

Cicindela (Ifasina) discrete sikhimensis : Mandl 1982: 64, 65.
Cylindera (Ifasina) sikhimensis : Jaskula 2008: 33.

##### New records.

Xizang, Motuo, 2 km SE of Beibeng, 29°14'8"N, 95°9'31"E, 843 m, light trap, leg. H.B. Liang, 29.vii.2012, 4 males, 9 females (IZCAS).

##### Distribution.

China (Xizang), India, Myanmar.

##### Remarks.

New state record for China and new provincial record for Xizang.

### ﻿Genus *Lophyra* Motschulsky, 1859

**Subgenus Spilodia Rivalier, 1961**

#### ﻿ Lophyra (Spilodia) lineifrons

Taxon classificationAnimaliaColeopteraCicindelidae

(Chaudoir, 1865)

E7B61142-B784-5B85-AFDE-E78175DBE675

Figs 6F , 8A

Cicindela lineifrons : Chaudoir 1865: 39, 62.
Lophyra (Spilodia) lineifrons : Shook and Wiesner 2006: 17; Wu 2011: 28.

##### Published data.

Yunnan (Shook and Wiesner 2006: 17; Wu 2011: 28).

**Figure 8.**
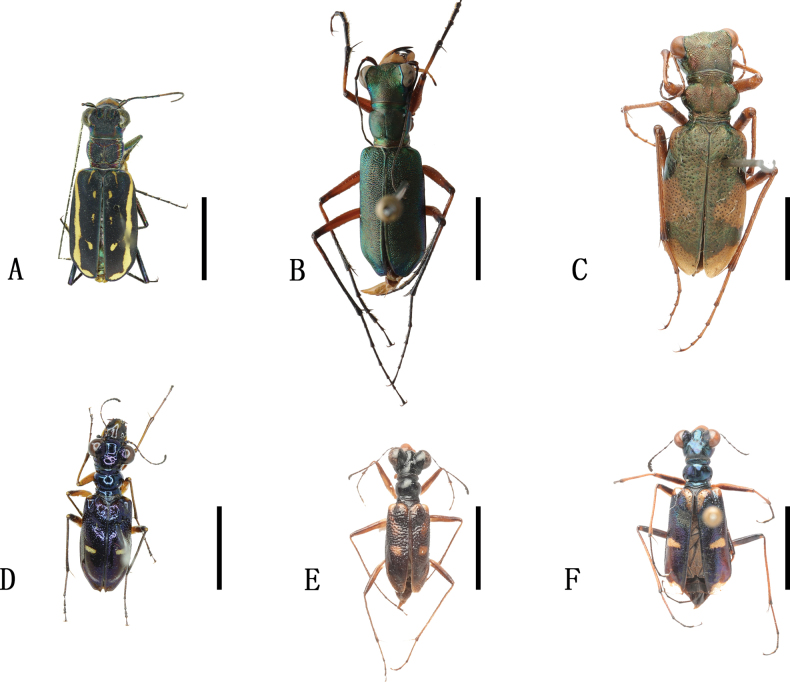
Habitus photographs **A**Lophyra (Spilodia) striolata dorsolineolata (Chevrolat, 1845) **B***Heptodontapulchella* (Hope, 1831) **C***Pronyssiformiaexcoffieri* (Fairmaire, 1897) **D***Theratesbiserratus* Tan, Mo & Liang, 1991 **E***Theratesguangdongensis* Wiesner, 2016 **F***Therateshunanensis* Matalin & Wiesner, 2023. Scale bars: 5 mm.

##### New records.

Guangxi, Ningming, Longrui, 180 m, leg. F.S. Li, 18.v.1984, 1 female (EMCAU).

##### Distribution.

China (Guangxi, Yunnan), Nepal, Bangladesh, India, Myanmar, Thailand, Cambodia, Laos, Vietnam, Malaysia.

##### Remarks.

New provincial record for Guangxi.

#### ﻿ Lophyra (Spilodia) striolatadorsolineolata

Taxon classificationAnimaliaColeopteraCicindelidae

(Chevrolat, 1845)

1EBCBF2F-0681-5ECD-80F8-E57EDF385903

Figs 8A , 9B

Cicindela dorsolineolata : Chevrolat 1845: 95, 96.
Lophyra (Spilodia) striolata dorsolineolata : Shook and Wiesner 2006: 17; Wu 2011: 28; Wiesner et al. 2017: 56.

##### Published data.

Beijing (Shook and Wiesner 2006: 17; Wu 2011: 28; Wiesner et al. 2017: 56), Hebei (Wu 2011: 28; Wiesner et al. 2017: 56), Shandong (Shook and Wiesner 2006: 17; Wu 2011: 28; Wiesner et al. 2017: 56), Jiangsu (Shook and Wiesner 2006: 17; Wu 2011: 28; Wiesner et al. 2017: 56), Zhejiang (Shook and Wiesner 2006: 17; Wu 2011: 28; Wiesner et al. 2017: 56), Anhui (Wu 2011: 28, Wiesner et al. 2017: 56), Jiangxi (Shook and Wiesner 2006: 17; Wu 2011: 28; Wiesner et al. 2017: 56), Fujian (Shook and Wiesner 2006: 17; Wu 2011: 28; Wiesner et al. 2017: 56), Henan (Shook and Wiesner 2006: 17; Wu 2011: 28; Wiesner et al. 2017: 56), Hubei (Shook and Wiesner 2006: 17; Wu 2011: 28; Wiesner et al. 2017: 56), Hunan (Shook and Wiesner 2006: 17; Wu 2011: 28; Wiesner et al. 2017: 56), Guangdong (Shook and Wiesner 2006: 17; Wu 2011: 28; Wiesner et al. 2017: 56), Guangxi (Wu 2011: 28; Wiesner et al. 2017: 56), Hainan (Shook and Wiesner 2006: 17; Wu 2011: 28; Wiesner et al. 2017: 56), Yunnan (Shook and Wiesner 2006: 17; Wu 2011: 28; Wiesner et al. 2017: 56), Guizhou (Wu 2011: 28; Wiesner et al. 2017: 56), Sichuan (Wu 2011: 28; Wiesner et al. 2017: 56), Taiwan (Shook and Wiesner 2006: 17; Wu 2011: 28; Wiesner et al. 2017: 56).

**Figure 9.**
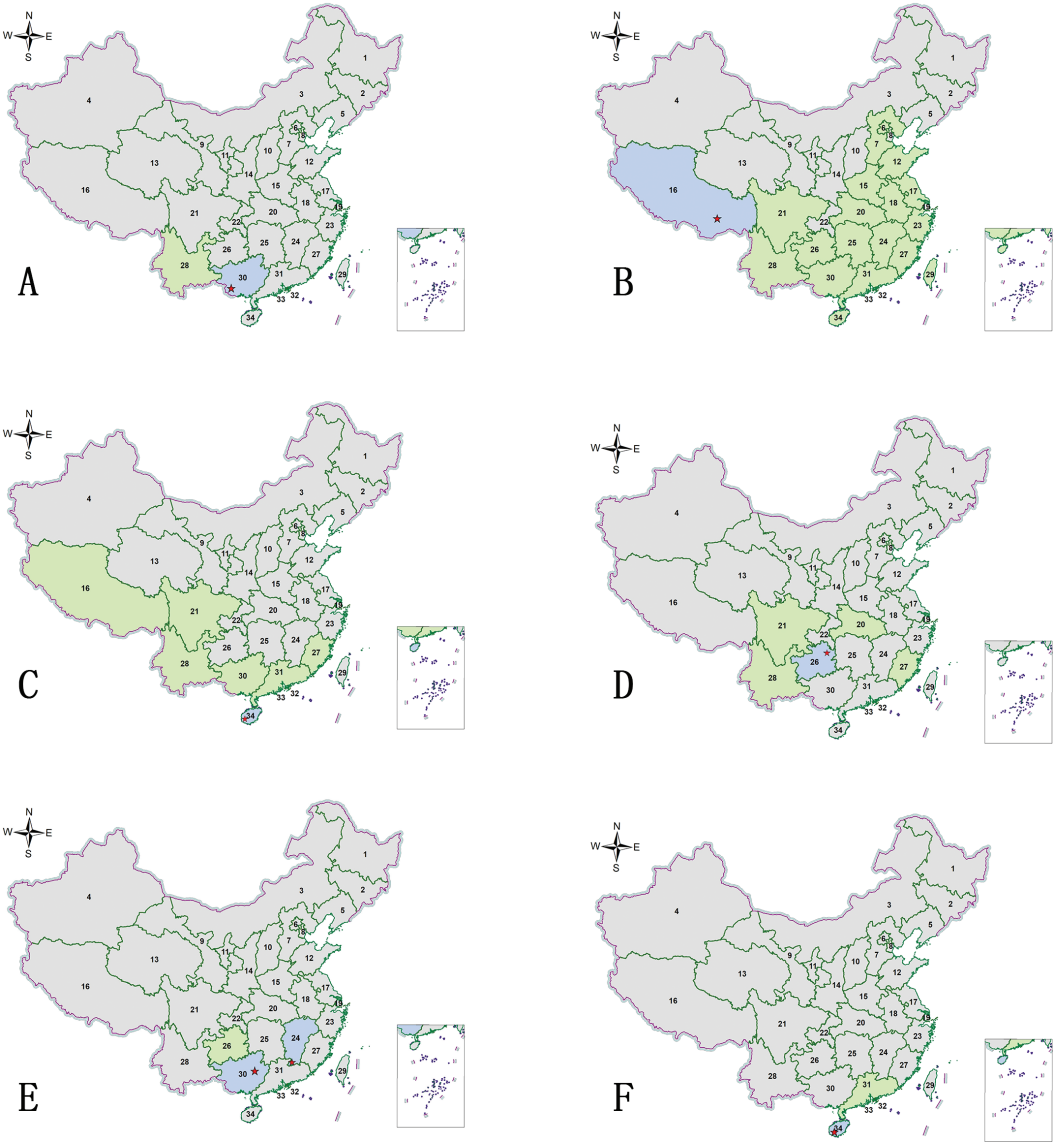
Distribution maps. Green indicates records with previously known distribution based on published data, blue indicates new records, red stars indicate the collection sites of the examined specimens **A**Lophyra (Spilodia) lineifrons (Chaudoir, 1865) **B**Lophyra (Spilodia) striolata dorsolineolata (Chevrolat, 1845) **C***Heptodontapulchella* (Hope, 1831) **D***Pronyssiformiaexcoffieri* (Fairmaire, 1897) **E***Theratesbiserratus* Tan, Mo & Liang, 1991 **F***Theratesguangdongensis* Wiesner, 2016.

##### New records.

Xizang, Beibeng, 2 km from Highway Gelin, 29°14'56"N, 95°11'20"E, 1013 m, leg. J.W. Jiang, 29.vii.2019, 3 males, 5 females (IZCAS).

##### Distribution.

China (Xizang, Beijing, Hebei, Shandong, Zhejiang, Jiangsu, Anhui, Jiangxi, Fujian, Henan, Hubei, Hunan, Guangdong, Guangxi, Hainan, Yunnan, Guizhou, Sichuan, Taiwan), Japan, Vietnam, Indonesia, Philippines.

##### Remarks.

New provincial record for Xizang.

### ﻿Subtribe Dromicina Thomson, 1859

**Genus *Heptodonta* Hope, 1838**

#### ﻿ Heptodonta pulchella

Taxon classificationAnimaliaColeopteraCicindelidae

(Hope, 1831)

518B0CF9-9779-5FD2-BB6F-4845F22AC96C

Figs 8B , 9C

Cicindela pulchella : Hope 1831: 21.
Heptodonta pulchella : Wu 2011: 28; Wiesner and Geiser 2016: 82; Görn 2020: 48.

##### Published data.

Fujian (Wu 2011: 28; Wiesner and Geiser 2016: 82; Görn 2020:51), Guangdong (Görn 2020:51), Guangxi (Görn 2020:51), Yunnan (Wu 2011: 28; Wiesner and Geiser 2016: 82; Görn 2020:51), Sichuan (Görn 2020:51), Xizang (Wu 2011: 28; Wiesner and Geiser 2016: 82; Görn 2020:51), Macao (Wu 2011: 28; Wiesner and Geiser 2016: 82).

##### New records.

Hainan, Jianfengling, Roadside of Tianchi, 18°43'44"N, 108°53'9"E, 1000 m, leg. H.B. Liang, 4.v.2007, 1 male, 2 females (IZCAS).

##### Distribution.

China (Hainan, Fujian, Guangdong, Guangxi, Yunnan, Sichuan, Xizang, Macao), Nepal, India, Myanmar, Thailand, Laos, Vietnam.

##### Remarks.

New provincial record for Hainan.

### ﻿Genus *Pronyssiformia* Horn, 1929

#### ﻿ Pronyssiformia excoffieri

Taxon classificationAnimaliaColeopteraCicindelidae

(Fairmaire, 1897)

DCC94D52-9E6E-5FD9-BBF7-E03A698A3A0B

Figs 8C , 9D

Cicindela excoffieri : Fairmaire 1897: 14.
Pronyssiformia excoffieri : Horn 1929: 5; Shook and Wiesner 2006: 20; Wu 2011: 31.

##### Published data.

Fujian (Shook and Wiesner 2006: 20; Wu 2011: 31), Hubei (Shook and Wiesner 2006: 20; Wu 2011: 31), Sichuan (Horn 1929: 6; Shook and Wiesner 2006: 20; Wu 2011: 31), Yunnan (Fairmaire 1897:14; Shook and Wiesner 2006; Wu 2011: 31).

##### New records.

Guizhou, Mount Fanjing, Huguosi, 27°54'44"N, 108°38'37"E, 1350 m, leg. Q.Z. Song, 3.viii.2001, 1 female (IZCAS).

##### Distribution.

China (Guizhou, Fujian, Hubei, Sichuan, Yunnan).

##### Remarks.

New provincial record for Guizhou.

### ﻿Subtribe Theratina Horn, 1893

**Genus *Therates* Latreille, 1816**

#### ﻿ Therates biserratus

Taxon classificationAnimaliaColeopteraCicindelidae

Tan, Mo & Liang, 1991

2372F4D9-FAF2-56A2-8480-E62C2FA87D0E

Figs 8D , 9E

Therates biserratus : Tan et al. 1991: 243; Matalin and Wiesner 2023: 414, 415.

##### Published data.

Guizhou (Jiangkou; Yinjiang) (Tan et al. 1991: 243; Matalin and Wiesner 2023: 414, 415).

##### New records.

Guangxi, Jinxiu, Luoxiang, 400 m, leg. D.C. Yuan, 14.v.1999, 1 male (IZCAS); Jiangxi, Longnan, Mount Jiulian, leg. Y.W. Zhang, 14.vi.1975, 1 female (IZCAS).

##### Distribution.

China (Guangxi, Jiangxi, Guizhou).

##### Remarks.

New provincial record for Guangxi and Jiangxi.

#### ﻿ Therates guangdongensis

Taxon classificationAnimaliaColeopteraCicindelidae

Wiesner, 2016

78999C14-BA5B-5139-8922-A5DAE71BCCDA

Figs 8E , 9F

Therates guangdongensis : Wiesner 2016: 131, 132.

##### Published data.

Guangdong (Yinnah Shan) (Wiesner 2016: 131,132).

##### New records.

Hainan, Baisha County, Nanmaola, leg. X.L. Huang, 13.v.2009, 1 male (IZCAS).

##### Distribution.

China (Guangdong, Hainan).

##### Remarks.

New provincial record for Hainan.

#### ﻿ Therates hunanensis

Taxon classificationAnimaliaColeopteraCicindelidae

Matalin & Wiesner, 2023

C5579BEC-D858-5A08-9533-C5F36548D4C1

Figs 8F , 11A

Therates hunanensis : Matalin and Wiesner 2023: 415.

##### Published data.

Hunan (Dong’an; Chengbu) (Matalin and Wiesner 2023: 415).

##### New records.

Chongqing, Mount Simian, 28°35–37'N, 106°23–24'E, 1190 m, leg. Z.H. Yang, 5.vii.2008, 1 male (IZCAS).

##### Distribution.

China (Chongqing, Hunan).

##### Remarks.

New provincial record for Chongqing.

#### ﻿ Therates probsti

Taxon classificationAnimaliaColeopteraCicindelidae

Wiesner, 1988

141366D7-96D3-5B73-B812-3C16FDB9ECFF

Figs 10A , 11B

Therates probsti : Wiesner 1988:20.

##### New records.

Xizang, Motuo, Yarang Hydropower Station, 29°15'54"N, 95°14'43"E, 850 m, leg. H.B. Liang, 1.viii.2019, 1 female (IZCAS).

**Figure 10.**
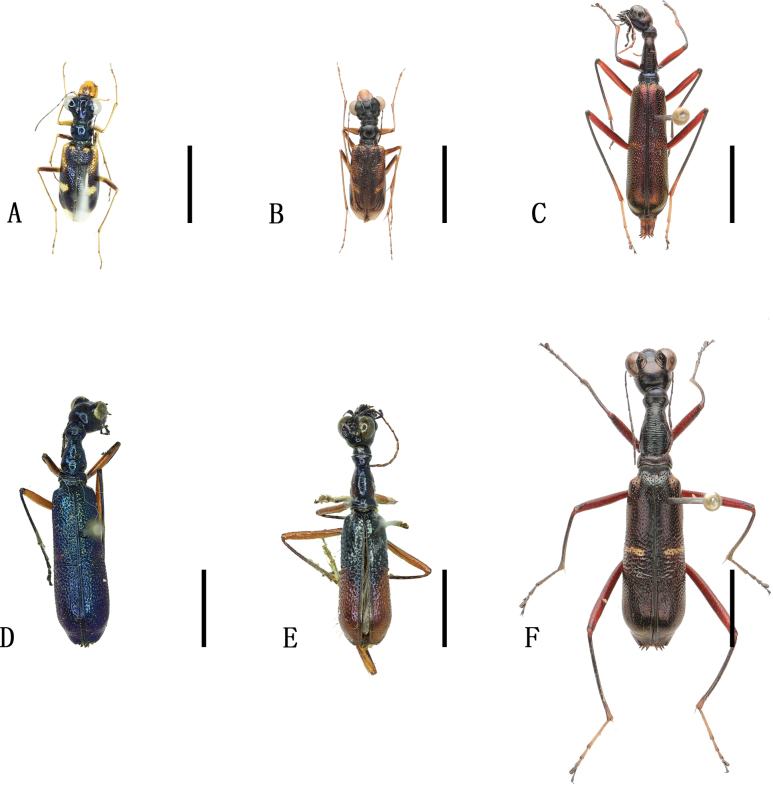
Habitus photographs **A***Theratesprobsti* Wiesner, 1988 **B***Theratesturnai* Wiesner, 2015 **C**Neocollyris (Isocollyris) grandivadosa (Horn, 1935) **D**Neocollyris (Neocollyris) saphyrina (Chaudoir, 1850) **E**Neocollyris (Pachycollyris) bicolor (Horn, 1902) **F**Neocollyris (Pachycollyris) mouhotii nagaii Naviaux & Sawada, 1992. Scale bars: 5 mm.

##### Distribution.

China (Xizang), Laos, Vietnam.

**Figure 11.**
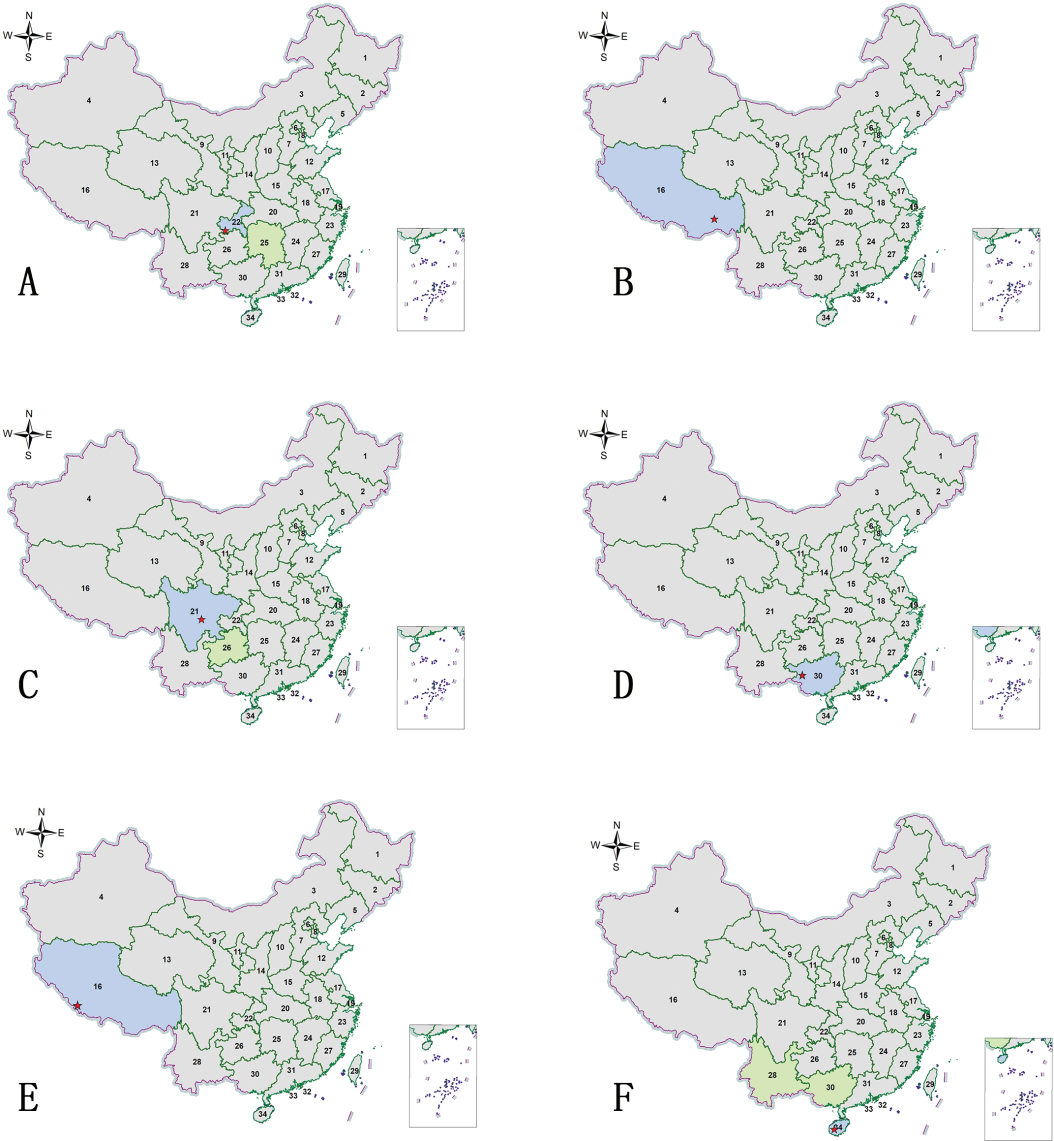
Distribution maps. Green indicates records with previously known distribution based on published data, blue indicates new records, red stars indicate the collection sites of the examined specimens **A***Therateshunanensis* Matalin & Wiesner, 2023 **B***Theratesprobsti* Wiesner, 1988 **C***Theratesturnai* Wiesner, 2015 **D**Neocollyris (Isocollyris) grandivadosa (Horn, 1935) **E**Neocollyris (Neocollyris) saphyrina (Chaudoir, 1850) **F**Neocollyris (Pachycollyris) bicolor (Horn, 1902).

##### Remarks.

New state record for China and new provincial record for Xizang.

#### ﻿ Therates turnai

Taxon classificationAnimaliaColeopteraCicindelidae

Wiesner, 2015

17BAE6D7-18CA-502B-B154-691B1B1F17DC

Figs 10B , 11C

Therates turnai : Wiesner 2015: 44–47.

##### Published data.

Guizhou (Xianheping) (Wiesner 2016: 44–47).

##### New records.

Sichuan, Mount Emei, Xixinsuo, 29°34'30"N, 103°22'26"E, 1300 m, day collecting, leg. H.B. Liang, 10–15.viii.2012, 1 male (IZCAS).

##### Distribution.

China (Guizhou, Sichuan).

##### Remarks.

New provincial record for Sichuan.

### ﻿Tribe Collyridini Brullé, 1834

**Subtribe Collyridini Brullé, 1834**

**Genus *Neocollyris* Horn, 1901**

**Subgenus Isocollyris Naviaux, 1994**

#### ﻿ Neocollyris (Isocollyris) grandivadosa

Taxon classificationAnimaliaColeopteraCicindelidae

(Horn, 1935)

23CB879F-D324-5C3F-98F3-E96B21A7C58F

Figs 10C , 11D

Collyris aureofusca grandi-vadosa : Horn 1935: 50, 51.
Neocollyris (Isocollyris) grandivadosa : Naviaux 2004: 74, 75, 76.

##### New records.

Guangxi, Napo County, 23°43'33"N, 106°49'30"E, 111 m, hand collected, leg. Y. Wang, 16.v.2021, 1 male, 1 female (IZCAS).

##### Distribution.

China (Guangxi), Vietnam.

##### Remarks.

New state record for China and new provincial record for Guangxi.

### Subgenus ﻿Neocollyris Horn, 1901

#### ﻿ Neocollyris (Neocollyris) saphyrina

Taxon classificationAnimaliaColeopteraCicindelidae

(Chaudoir, 1850)

0CE91972-50F1-59BC-AD70-80A4102278E8

Figs 10D , 11E

Neocollyris (Neocollyris) saphyrina : Li and Chen 1993: 102.

##### Published data.

?Yunnan (Li and Chen 1993: 102), ?Sichuan (Xichang) (Li and Chen 1993: 102).

##### New records.

Xizang, Shigatse, Gyirong, 2400 m, leg. F.S. Huang, 22.vii.1975, 1 female (IZCAS).

##### Distribution.

China (Xizang, ?Yunnan, ?Sichuan), India, Myanmar, Laos, Nepal, Bhutan, Bangladesh, Thailand, Indonesia.

##### Remarks.

Gyirong borders Nepal, from where *N.saphyrina* was previously known. Shook and Wiesner (2006) indicated that records included by Li and Chen (1993) for N. (N.) saphyrina in Yunnan and Sichuan (Xichang) required confirmation. The location reported here, Gyirong, is far from Yunnan and Sichuan, and the closest confirmed distribution of N. (N.) saphyrina to Yunnan and Sichuan is in Cambodia and Laos, but with some similar species present in this area, the records for Yunnan and Sichuan still need to be re-examined. We restore the record of N. (N.) saphyrina for China with a new provincial record for Xizang.

### Subgenus ﻿Pachycollyris Naviaux, 1995

#### ﻿ Neocollyris (Pachycollyris) bicolor

Taxon classificationAnimaliaColeopteraCicindelidae

(Horn, 1902)

1D0F3CB1-0D4D-5D32-B754-68F06EED7F26

Figs 10E , 11F

Collyris bicolor : Horn 1902: 70.
Neocollyris (Pachycollyris) bicolor : Wu 2011: 30; Wiesner and Geiser 2016: 77.

##### Published data.

Guangxi (Wu 2011: 30; Wiesner and Geiser 2016: 77), Yunnan (Wu 2011: 30; Wiesner and Geiser 2016: 77).

##### New records.

Hainan, Wuzhishan, Fanyang Town, leg. G. Ros, 1.vii.1956, 1 female (IZCAS).

##### Distribution.

China (Hainan, Guangxi, Yunnan), Laos, Vietnam.

##### Remarks.

New provincial record for Hainan.

#### ﻿ Neocollyris (Pachycollyris) mouhotiinagaii

Taxon classificationAnimaliaColeopteraCicindelidae

Naviaux & Sawada, 1992

D4481CB0-AC7A-50FF-AC02-768F77BB4861

Figs 10F , 13A

Neocollyris mouhotii nagaii : Naviaux and Sawada 1992: 46, 47, 48.
Neocollyris (Pachycollyris) mouhotii nagaii : Naviaux 1995: 267.

##### New records.

Hainan, Baisha County, Yinggeling, 18°59'28"N, 109°20'18"E, leg. M.Y. Lin, 14.vi.2010, 1 female (IZCAS).

##### Distribution.

China (Hainan), Vietnam.

##### Remarks.

New state record for China and new provincial record for Hainan.

#### ﻿ Neocollyris (Pachycollyris) sawadai

Taxon classificationAnimaliaColeopteraCicindelidae

Naviaux, 1991

B148B23B-A648-5B7C-A7C2-606A559620FB

Figs 12A , 13B

Neocollyris sawadai : Naviaux 1991a: 222.

##### New records.

Yunnan, Pu’er, Simao, day collecting, leg. H.L. Han and M.J. Qi, 15–19.vii.2009, 1 female (NEFU).

**Figure 12.**
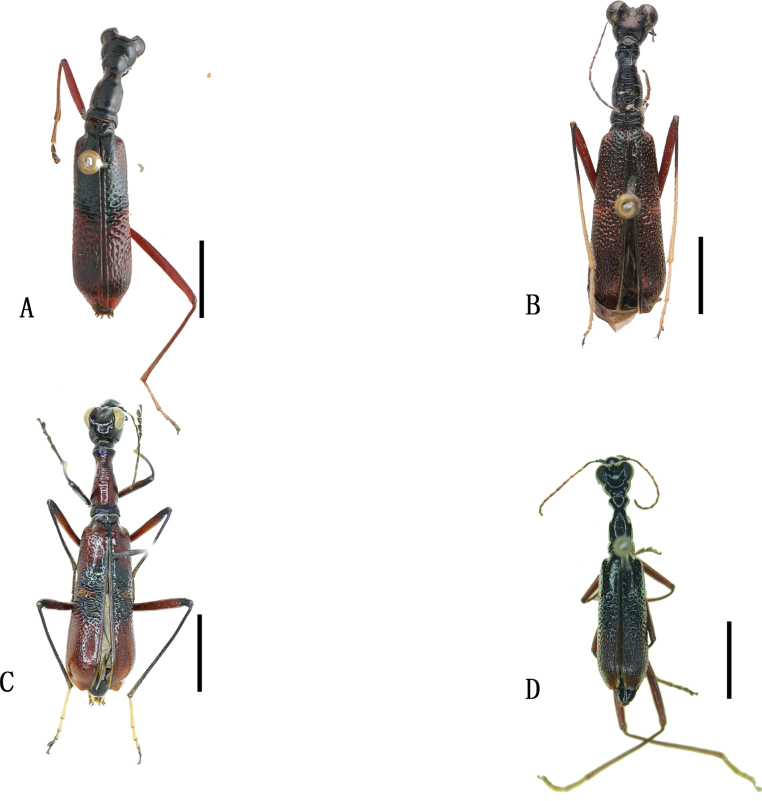
Habitus photographs **A**Neocollyris (Pachycollyris) sawadai Naviaux, 1991 **B**Neocollyris (Pachycollyris) strangulata Naviaux, 1991 **C**Neocollyris (Pachycollyris) tricolor Naviaux, 1991 **D**Neocollyris (Pachycollyris) vitalisi (Horn, 1924). Scale bars: 5 mm.

##### Distribution.

China (Yunnan), Vietnam.

**Figure 13.**
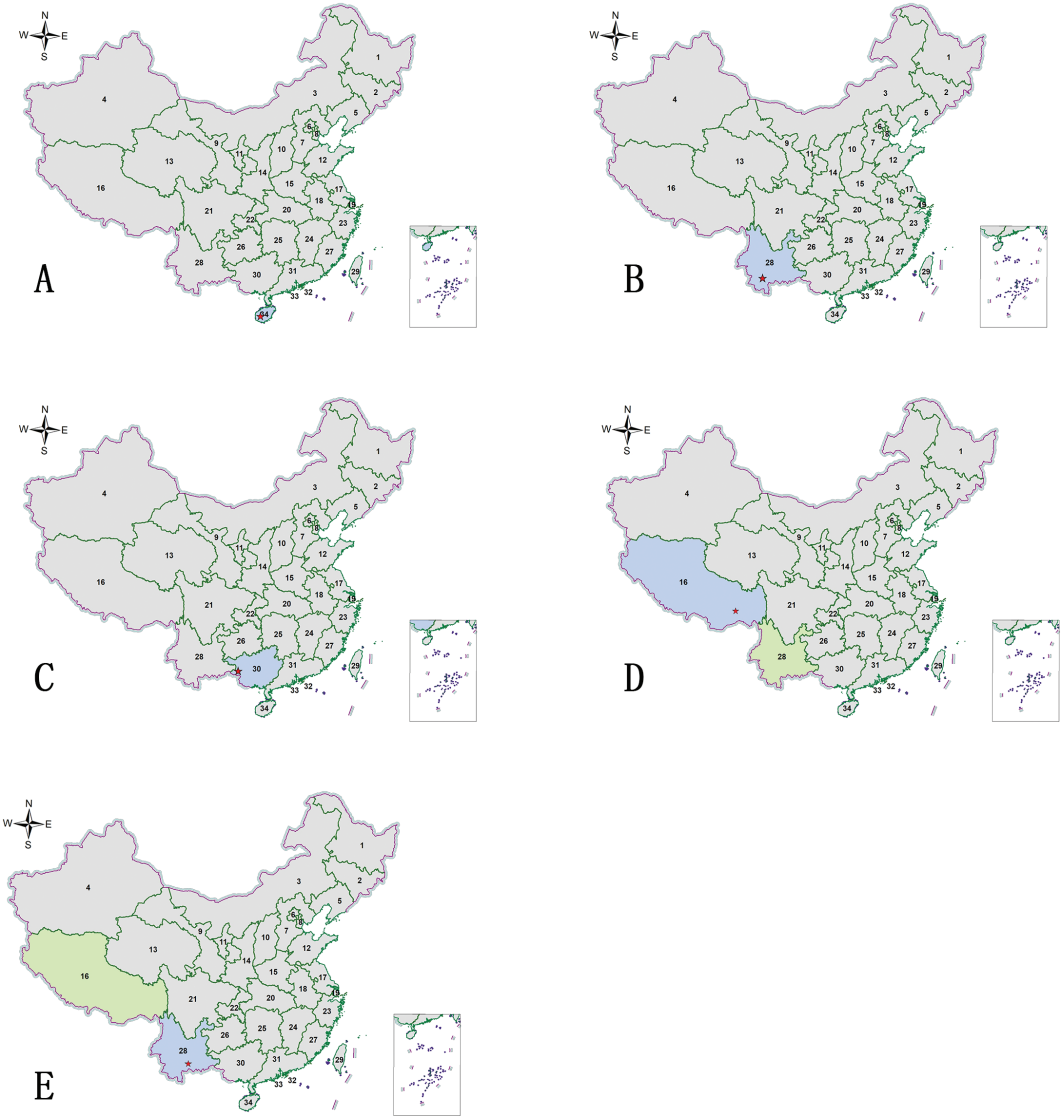
Distribution maps. Green indicates records with previously known distribution based on published data, blue indicates new records, red stars indicate the collection sites of the examined specimens **A**Neocollyris (Pachycollyris) mouhotii nagaii Naviaux & Sawada, 1992 **B**Neocollyris (Pachycollyris) sawadai Naviaux, 1991 **C**Neocollyris (Pachycollyris) strangulata Naviaux, 1991 **D**Neocollyris (Pachycollyris) tricolor Naviaux, 1991 **E**Neocollyris (Pachycollyris) vitalisi (Horn, 1924).

##### Remarks.

New state record for China and new provincial record for Yunnan.

#### ﻿ Neocollyris (Pachycollyris) strangulata

Taxon classificationAnimaliaColeopteraCicindelidae

Naviaux, 1991

D539AF2F-653D-55A0-8505-ADA9A6720777

Figs 12B , 13C

Neocollyris strangulata : Naviaux 1991b: 276, 277.
Neocollyris (Pachycollyris) strangulata : Naviaux 1995: 263, 264.

##### New records.

Guangxi, Jingxi City, Diding Nature Reserve, 23°6'47"N, 105°58'40"E, leg. S.Y. Zhou and J.H. Huang, 9.viii.2010, 1 female (IZCAS).

##### Distribution.

China (Guangxi), Laos, Vietnam.

##### Remarks.

New state record for China and new provincial record for Guangxi.

#### ﻿ Neocollyris (Pachycollyris) tricolor

Taxon classificationAnimaliaColeopteraCicindelidae

Naviaux, 1991

0A6E2BE7-1774-50A7-98CC-464C7AA9743B

Figs 12C , 13D

Neocollyris tricolor : Naviaux 1991c: 19, 20.
Neocollyris (Pachycollyris) tricolor : Wu and Shook 2007: 39; Wu 2011: 31.

##### Published data.

Yunnan (Lincang) (Wu and Shook 2007:39; Wu 2011: 31).

##### New records.

Xizang, Beibeng, 2 km from Highway Gelin, 29°14'56"N, 95°11'20"E, 1013 m, leg. J.W. Jiang, 29.vii.2019, 1 female (IZCAS).

##### Distribution.

China (Xizang, Yunnan), Myanmar, Thailand, Laos, Vietnam.

##### Remarks.

New provincial record for Xizang.

#### ﻿ Neocollyris (Pachycollyris) vitalisi

Taxon classificationAnimaliaColeopteraCicindelidae

(Horn, 1924)

6D789420-2680-543B-A6F9-C16D174371FC

Figs 12D , 13E

Neocollyris (Pachycollyris) feai vitalisi : Naviaux 1995: 256, 257.
Neocollyris (Pachycollyris) vitalisi : Cassola 2005: 15; Wiesner and Geiser 2016: 78.

##### Published data.

Xizang (Wiesner and Geiser 2016: 78).

##### New records.

Yunnan, Mount Huanglian, Beiluo, 22°44'8"N, 102°18'27"E, 1277 m, day collecting, leg. G.Z. Zhong and L.K. Zhang, 09–15.IX.2016, 1 female (KIZCAS).

##### Distribution.

China (Yunnan, Xizang), Myanmar, Thailand, Laos, Vietnam.

##### Remarks.

New provincial record for Yunnan.

## ﻿Discussion

**Figure 14.**
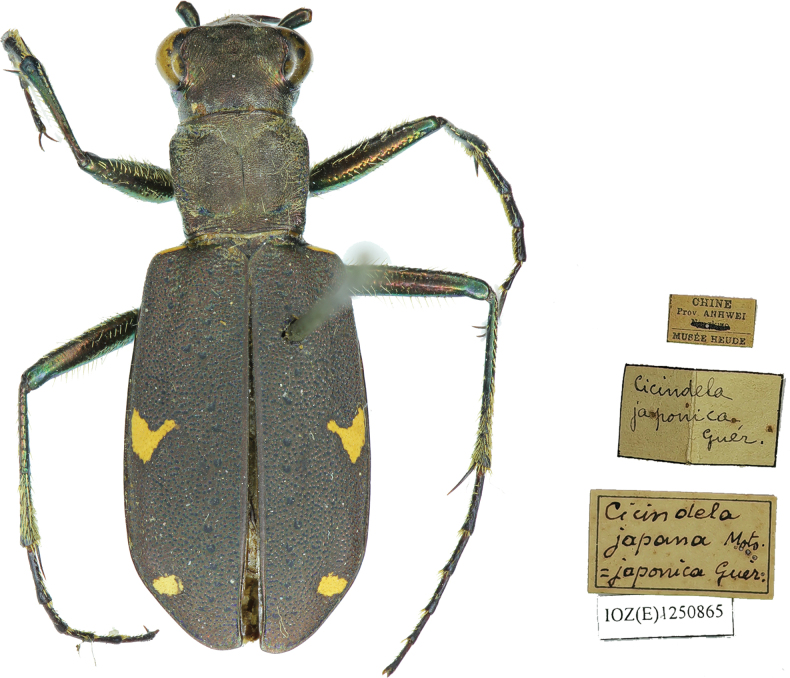
Cicindela (Cicindela) japana Motschulsky, 1858. Habitus and specimen labels.

During our investigation of Chinese collections, we encountered a single specimen of Cicindela (Cicindela) japana Motschulsky, 1858 [Anhui, China = CHINE, Prov. ANHWEI-printed label] (Fig. 14). Cicindela (C.) japana is only known from Japan, where it occurs on the four main islands and several smaller adjacent islands. One specimen deposited in a museum collection in Vladivostok was collected on Kunashir Island and supposedly blown there from Hokkaido by strong winds (A.V. Matalin pers. comm.). Previous records of C. (C.) japana from China and South Korea that could be examined by us all referred to misidentified specimens of C. (C.) sachalinensis raddei. Thus, it seems unlikely that C. (C.) japana occurs in China, especially in an inland province far from Japan. Given the age of this specimen, and the absence of labels indicating the collector and date, we suspect it may have been mislabeled.

With the new records presented here, the tiger beetle fauna of China now includes 208 species, 71 (34%) of which are endemic. Five of the new records reported here are from the Chayu and Motuo, regions in the southeast of Xizang. This area is located at the junction of the Hengduan Mountains and the Himalayas and is surrounded by many biogeographical barriers. These five species were previously known from adjacent regions and countries, including Yunan Province, Sichuan Province, Myanmar, and India, from which they may have dispersed. More research on the tiger beetle diversity in this region, the southeast of Xizang, is needed and has great potential to broaden our understanding of the biogeography of tiger beetles within China.

Recently, Matalin et al. (2024) restored Apterodela (Apterodela) bivirgulata (Fairmaire, 1889) as a separate species. Before the reclassification, Apterodela (Apterodela) lobipennis (Bates, 1888) was regarded as one of the common tiger beetles in China, with many specimens preserved in collections. All these specimens need to be re-examined to determine their exact classification. Therefore, additional specimens and photographic records will facilitate the progress of identification.

Furthermore, we report new provincial records of *Theratesbiserratus* Tan, Mo & Liang, 1991 and *Therateshunanensis* Matalin & Wiesner, 2023 in China. *Theratesbiserratus* has been included in the List of Key Protected Wild Animals in China since 2003, although it has not been considered as a separate species for a long time. According to the known distribution, *T.biserratus* may indeed be widely distributed in Nan Ling Area (24°00'–26°30'N, 110°–116°E). This may ultimately lead to reconsideration of the taxonomic status between these two species. Due to the limited number of specimens included in this and other studies (Matalin and Wiesner 2023), more specimens are needed to adequately determine their status.

To date, taxonomic studies on tiger beetles from Guangxi and Hainan are relatively scarce (Naviaux 2010; Xiong and Wiesner 2022). Here, we report nine new records from Guangxi and four new records from Hainan. We believe that the diversity of tiger beetles in these two provinces far exceeds the currently known records and deserves more attention from taxonomists.

Many of the specimens reported here are old specimens that have not been previously identified. There are likely numerous unidentified tiger beetle specimens in other institutions and museums in China, which hold considerable yet untapped data. We encourage others to recognize and utilize this potential and are willing to help in identification.

## Supplementary Material

XML Treatment for Abroscelis anchoralis anchoralis

XML Treatment for Apterodela (Apterodela) bivirgulatabivirgulata

XML Treatment for Callytron nivicinctum

XML Treatment for Calomera chloris

XML Treatment for Calomera plumigera scoliographa

XML Treatment for Cicindela (Cicindela) campestrispontica

XML Treatment for Cicindela (Cicindela) sachalinensisraddei

XML Treatment for Cicindela (Cicindela) transbaicalicahamifasciata

XML Treatment for Cosmodela separata

XML Treatment for Cylindera (Cylindera) obliquefasciata

XML Treatment for Cylindera (Eriodera) albopunctata

XML Treatment for Cylindera (Eugrapha) contortacontorta

XML Treatment for Cylindera (Eugrapha) elisaeelisae

XML Treatment for Cylindera (Eugrapha) sublaceratavicaria

XML Treatment for Cylindera (Ifasina) decolorata

XML Treatment for Cylindera (Ifasina) kaleeakaleea

XML Treatment for Cylindera (Ifasina) lesnei

XML Treatment for Cylindera (Ifasina) sikhimensis

XML Treatment for Lophyra (Spilodia) lineifrons

XML Treatment for Lophyra (Spilodia) striolatadorsolineolata

XML Treatment for Heptodonta pulchella

XML Treatment for Pronyssiformia excoffieri

XML Treatment for Therates biserratus

XML Treatment for Therates guangdongensis

XML Treatment for Therates hunanensis

XML Treatment for Therates probsti

XML Treatment for Therates turnai

XML Treatment for Neocollyris (Isocollyris) grandivadosa

XML Treatment for Neocollyris (Neocollyris) saphyrina

XML Treatment for Neocollyris (Pachycollyris) bicolor

XML Treatment for Neocollyris (Pachycollyris) mouhotiinagaii

XML Treatment for Neocollyris (Pachycollyris) sawadai

XML Treatment for Neocollyris (Pachycollyris) strangulata

XML Treatment for Neocollyris (Pachycollyris) tricolor

XML Treatment for Neocollyris (Pachycollyris) vitalisi

## ﻿Additional information

### Author ORCIDs

Ke-Yi Wang https://orcid.org/0009-0009-8915-9563

Fabian A. Boetzl https://orcid.org/0000-0001-5121-3370

Jürgen Wiesner https://orcid.org/0000-0002-7824-166X

### Data availability

All of the data that support the findings of this study are available in the main text.
